# Anthropomorphism to Zoonoses: Two Inevitable Consequences of Human–Animal Relationships

**DOI:** 10.3201/eid2112.AC2112

**Published:** 2015-12

**Authors:** Byron Breedlove, Paul M. Arguin

**Affiliations:** Centers for Disease Control and Prevention, Atlanta, Georgia, USA

**Keywords:** art science connection, emerging infectious diseases, art and medicine, about the cover, anthropomorphism, Beauty and the Beast, Walter Crane, anthropomorphism to zoonoses: two inevitable consequences of human–animal relationships, zoonoses

**Figure:**
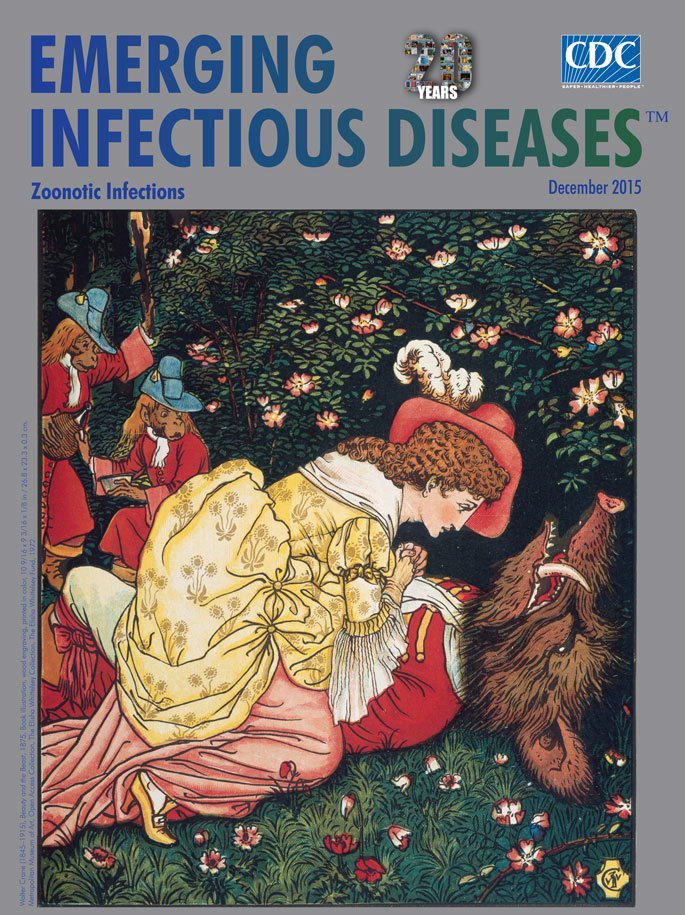
**Walter Crane (1845–1915), Beauty and the Beast, 1875. Book illustration, wood engraving, printed in colors, 10 9/16 × 9 3/16 × 1/8 in/26.8 × 23.3 × 0.3 cm.** Metropolitan Museum of Art, Open Access Collection, The Elisha Whittelsey Collection, The Elisha Whittelsey Fund, 1972

Many tales portray animals that mimic human behavior and characteristics by conversing, walking erectly, dressing in clothing, and inhabiting houses. Across myriad cultures and throughout history, stories and myths that feature anthropomorphism have helped us understand and relate to the natural world. This device is especially common, even expected, in children’s literature.

Among the most enduring anthropomorphic stories is *Beauty and the Beast,* retold and reimagined in various printed versions, films, and plays. French author Gabrielle-Suzanne Barbot de Villeneuve (c. 1695−1755) is credited with creating the first version of the tale we know as Beauty and the Beast (*La Belle et la Bête*). Her 100-plus page story features a savage beast and various subplots, setting it apart from most subsequent versions; those derive from the 1767 abridged narrative by Jeanne-Marie Leprince de Beaumont, which depicts a kinder, gentler beast.

This month’s cover image comes from a popular version of *Beauty and the Beast* by Walter Crane (1845−1915), published by John Lane in 1875. Crane’s wood engravings used for the illustrations in this edition incorporated design elements from Japanese prints, classical sculpture, and tapestry. Crane has been recognized as “the most prolific and influential children’s book creator of his generation” and because of his later success in other artistic endeavors as “one of the most ambitious British artists of the later nineteenth century.”

This colorful, meticulously detailed illustration depicts a young woman, Beauty, dressed in prim Victorian clothing, administering to the dying Beast, a boar that has an elongated snout and tusks, but is dressed in a handsome red suit, lying in a field of flowers and surrounded by a dense forest. Two smartly dressed monkeys toting a fiasco of wine, “who always waited upon her with all the attention and respect that officers of a royal household are accustomed to pay to queens,” watch with concern as the Beast’s life hangs in the balance. Beauty, of course, saves the Beast and breaks the spell, restoring him to a handsome prince and leading to the “happily ever after” ending.

*Beauty and the Beast* offers a lens for viewing humankind’s relationship and interactions with the other animals on the planet. Anthropomorphism—which requires the suspension of disbelief to pretend the impossible is real—is inevitable, considering that people have lived in close proximity to domesticated and wild animals for millennia. We rely on “beasts” for sustenance, transportation, and labor; as surrogates in our scientific and medical experiments; and as companions, protectors, and entertainers. We have a protracted history of both legal and illegal hunting and trading of wildlife, and we have long practiced and animal idolatry and worship.

Zoonoses are also an inevitable consequence of human−animal relationships and interactions. Zoonotic diseases can be caused by organisms such as viruses, bacteria, parasites, and fungi, and it is estimated that more than 60% of infectious diseases of humans are spread from animals. Knowing which animals could have zoonotic diseases proves challenging because both domesticated animals and wildlife may appear and act healthy and yet be carrying lethal pathogens.

With this knowledge, we can view the painting as a study in potential zoonotic exposures to poor Beauty. Is Beast really dying for want of the love of a beautiful maiden? Or is he gasping for breath due to his Nipah virus or swine influenza infection? One can almost hear Beast’s rumbling cough as Beauty becomes infected by droplet transmission at such close proximity. And what about the healthy-appearing monkey attendants poised to pour a cup of wine? Southeast Asian macaques may be asymptomatically infected with herpes B virus or *Plasmodium knowlesi* parasites. Utensils shared with a monkey would be a convenient fomite for herpes B virus, which can be transmitted through monkey saliva. The woodland setting is also the ideal habitat for arboreal *Anopheles* mosquitoes that have been implicated in the transmission of malaria from monkeys to humans.

The One Health concept recognizes that the health of humans is connected to the health of animals and the environment. Interaction with animals is an integral part of our lives that works when we are mindful of the risks and take appropriate precautions when necessary.

## References

[1] Centers for Disease Control and Prevention. One Health [cited 2015 Oct 13]. http://www.cdc.gov/onehealth/about.html

[2] Centers for Disease Control and Prevention. Zoonotic diseases [cited 2015 Sep 29]. http://www.cdc.gov/onehealth/zoonotic-diseases.html

[3] Crane W. Beauty and the Beast. London; New York: George Routledge and Sons; 1875, p. 1–26 [cited 2015 Sep. 23]. https://archive.org/details/beautybeast00cra

[4] Delaney L. Walter Crane: a revolution in nursery picture books [cited 2015 Sep 22]. http://booksforkeeps.co.uk/issue/185/childrens-books/articles/other-articles/walter-crane-a-revolution-in-nursery-picture-books

[5] Ganea PA, Canfield CF, Simons-Ghafari K, Chou T. Do cavies talk? The effect of anthropomorphic picture books on children’s knowledge about animals. Front Psychol. 2014;5:283. 10.3389/fpsyg.2014.00283 24782793 PMC3989584

[6] Gooding R. Walter Crane’s toy books [cited 2015 Sep 22]. http://www.reading.ac.uk/web/FILES/special-collections/featurecrane.pdf

[7] Griswold J. The meanings of “Beauty and the Beast”: a handbook. Peterborough (Ontario, Canada): Broadview Press; 2004; p. 9–11, 16–7.

[8] Kruse H, Kirkemo A-M, Handeland K. Wildlife as source of zoonotic infections. Emerg Infect Dis. 2004;10:2067–72. 10.3201/eid1012.040707 15663840 PMC3323390

[9] Parashar UD, Sunn LM, Ong F, Mounts AW, Arif MT, Ksiazek TG, Case–control study of risk factors for human infection with a new zoonotic paramyxovirus, Nipah virus, during a 1998−1999 outbreak of severe encephalitis in Malaysia. J Infect Dis. 2000;181:1755–9. 10.1086/315457 10823779

[10] Zipes J. Fairy tale as myth. Myth as fairy tale. Lexington (Kentucky): The University Press of Kentucky; 1994. p. 24–5.

